# An Improved HPLC Method with the Aid of a Chemometric Protocol: Simultaneous Determination of Atorvastatin and Its Metabolites in Plasma

**DOI:** 10.3390/molecules18032469

**Published:** 2013-02-25

**Authors:** Milkica Crevar-Sakač, Zorica Vujić, Jasmina Brborić, Vesna Kuntić, Snežana Uskoković-Marković

**Affiliations:** 1 Department of Pharmaceutical Chemistry, University of Belgrade-Faculty of Pharmacy, Vojvode Stepe 450, Belgrade 11221, Serbia; 2 Department of Physical Chemistry, University of Belgrade-Faculty of Pharmacy, Vojvode Stepe 450, Belgrade 11221, Serbia; 3 Department of Analytical Chemistry, University of Belgrade-Faculty of Pharmacy, Vojvode Stepe 450, Belgrade 11221, Serbia

**Keywords:** atorvastatin (acid and lactone), *ortho*- and *para*-hydroxyatorvastatin, central composite design, Derringer’s desirability function, HPLC

## Abstract

The aim of the present study was to optimize a chromatographic method for the analysis of atorvastatin (acid and lactone forms), *ortho*- and *para*-hydroxyatorvastatin by using an experimental design approach. Optimization experiments were conducted through a process of screening and optimization. The purpose of a screening design is to identify the factors that have significant effects on the selected chromatographic responses, and for this purpose a full 2^3^ factorial design was used. The location of the true optimum was established by applying Derringer’s desirability function, which provides simultaneously optimization of all seven responses. The ranges of the independent variables used for the optimization were content of acetonitrile in mobile phase (60–70%), temperature of column (30–40 °C) and flow rate (0.8–1.2 mL min^−1^). The influences of these independent variables were evaluated for the output responses: retention time of first peak (*p*-hydroxyatorvastatin) and of last peak (atorvastatin, lactone form), symmetries of all four peaks and relative retention time of *p*-hydroxyatorvastatin. The primary goal of this investigation was establishing a new simple and sensitive method that could be used in analysis of biological samples. The method was validated and successfully applied for determination of atorvastatin (acid and lactone forms) and its metabolites in plasma.

## 1. Introduction

**Scheme 1:**
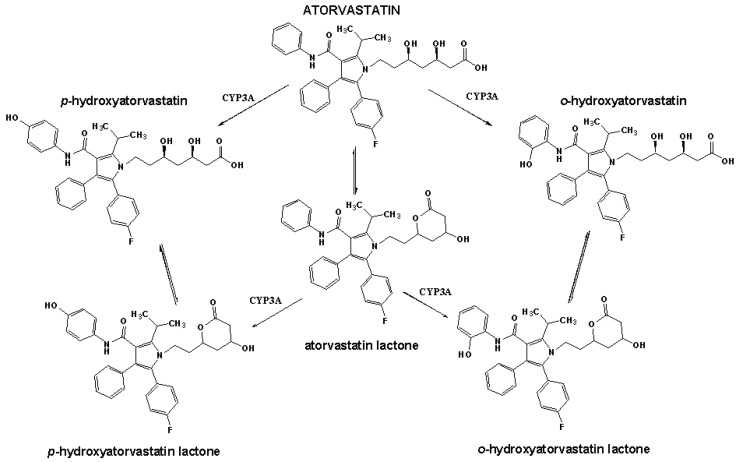
Structures of atorvastatin and its metabolites and the metabolic pathways of atorvastatin [2].

Atorvastatin, (3*R*,5*R*)-7-[2-(4-fluorophenyl)-3-phenyl-4-(phenylcarbamoyl)-5-propan-2-yl-pyrrol-1-yl]-3,5-dihydroxyheptanoic acid (abbreviated as ATO), is a member of the drug class known as statins which are used for lowering blood cholesterol. Like all statins, atorvastatin works by inhibiting HMG-CoA reductase, an enzyme found in liver tissue that plays a key role in production of cholesterol in the body. Atorvastatin is administered as the calcium salt of its active acid form. The primary proposed mechanism of atorvastatin metabolism is through cytochrome P450 3A4 hydroxylation to form active *ortho*- and *para*-hydroxylated metabolites [1]. About 70% of the total plasma HMG-CoA reductase inhibitory activity is accounted for by active metabolites (Scheme 1), adapted from [2].

To date, several methods have been published for quantification of atorvastatin and its metabolites in a biological matrix (plasma or serum), bulk drug, pharmaceuticals products, and aqueous samples. Various techniques employed include a derivative spectrophotometric method [1], UV spectroscopy [3,4], HPTLC [5], enzyme inhibition [6] with radioactivity detection [7], as well as a number of HPLC methods.

Some published papers have dealt with HPLC determination of atorvastatin alone [8], or in the presence of aspirin [9], ramipril and aspirin [10], nicotinic acid [11], amlodipine besylate [12], ezetimibe [13], ezetimibe and fenofibrate [14] and other statins [15,16]. For separation and quantification of atorvastatin and its impurities, a liquid chromatography method was also proposed [17]. A stability indicating method for simultaneous determination of atorvastatin, fenofibrat and their degradation products has been described by Kadav and Vora [18].

Rapid, sensitive and effective methods for determination of drugs and metabolites in biological fluids are desirable [19]. A literature survey revealed that the most often used method for the determination plasma levels of atorvastatin and its active and inactive metabolites is LC-MS/MS [20,21]. Liquid chromatography-tandem mass spectrometry (LC-MS/MS) is suitable since MS/MS detection is sensitive and enables effective elimination of interferences from endogenous components. For this reason most published methods for determination of ATO or both metabolites in biological fluids require a method of LC-MS/MS [22,23,24], especially because of the limitations of all previously mentioned instrumental methods.

High-performance liquid chromatography with ultra-violet or electrochemical detection methods typically has a higher limit of quantification and is usually time-consuming. Gas chromatography meets the required of limit of quantification but it needs complex derivatization steps. Enzyme inhibition assays are sensitive and easy to implement but are non-specific and do not provide any information on the metabolite concentrations.

To the best of our knowledge, currently there is no HPLC method employing optimization techniques for determination of atorvastatin and its metabolites in biological sample. This work is an attempt of establishing an HPLC method which would be simple and sensitive and could be an alternative method to LC/MS/MS.

The goal was conducted by using the optimization step accomplished by Derringer’s desirability function. The main advantage of such approach is simultaneous optimization of influencing factors and response variables which enables prediction of chromatographic retention and postulation of optimum conditions for separation.

## 2. Results and Discussion

**Figure 1:**
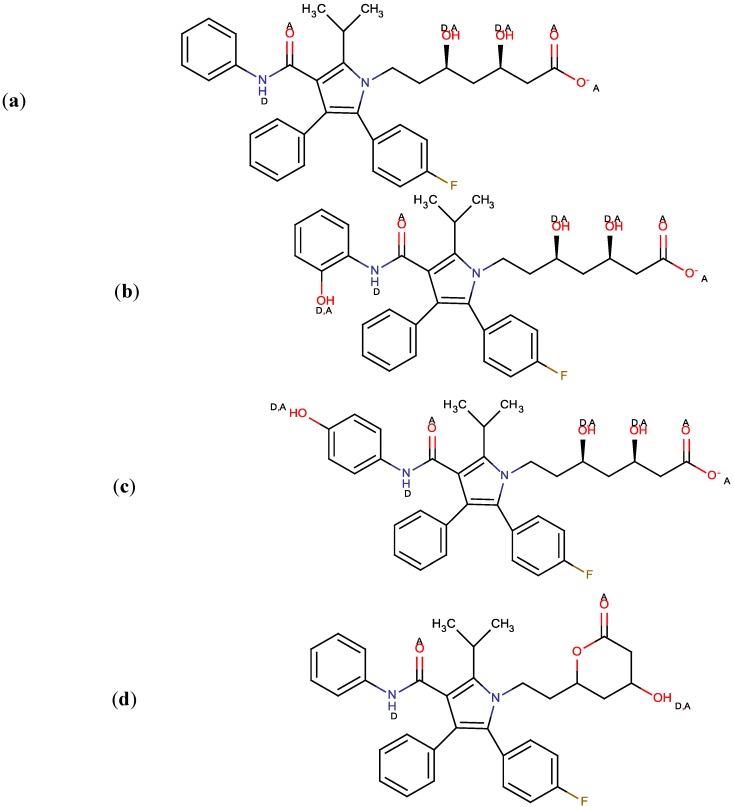
Proton acceptor (A) and proton donor groups (D) of atorvastatin (**a**), *ortho*- hydroxylated metabolite (**b**), *para*-hydroxylated metabolite (**c**) and atorvastatin lactone (**d**).

The presence of aromatic functional groups in the molecular structure of atorvastatin, such as phenyl and pyrol, makes a RP-HPLC method with PDA detection suitable for the determination. Since the RP-HPLC method is based on using a polar mobile phase, a complete description of the ionization profile of atorvastatin and its metabolites have been used for the evaluation of retention behavior and solubility. Considering the chemical structures and using MarvinSketch software, it is possible to establish a number of proton acceptor and donor groups (Figure 1a–d) at physiological pH (7.4) and to calculate Log P.

According to the obtained results, pKa values are: 4.33 (*p*-ATO); 4.33 and 8.76 (*o*-ATO), which makes these compounds soluble in a mixture of acetonitrile (ACN) and water, while ATO-l does not have an ionisable group and is soluble in acetonitrile. The degree of ionization of the drug strongly affects solubility and retention.

In accordance with pKa values, acetate buffer pH 4.6 was used for dilution of these compounds. At this pH, compounds are presented in ionisable and unionisable forms (approximately 50:50). By applying MarvinSketch software logP values were calculated: 5.39 for ATO; 5.08 for *o*-ATO and 6.05 for ATO-l. ATO acid is more lipid-soluble than its hydroxylated metabolites and less than the lactone form. Considering this fact, the order of elution was predictable: *p*-ATO; *o*-ATO, ATO; ATO-l.

Developing and optimizing isocratic HPLC methods is a complex procedure that requires simultaneous determination of several factors. An HPLC method could be optimized by trial and error methodology, but this approach is time consuming and concerns the influence of one factor at time on response, while the information about other factors as well as interaction between factors is not available. In order to optimize more than one response at a time the chemometric methods which includes factorial design [25] and response surface methodology [26] should be applied.

This paper deals with multiple response simultaneous optimizations using the Derringer’s desirability function for the development of a reversed-phase HPLC method for the simultaneous determination atorvastatin (acid and lactone forms), *o*- and *p*-hydroxyatorvastatin.

The selection of key factors examined for optimization was based on preliminary experiments and prior knowledge from literature. The factors selected for optimization process were: content of acetonitrile in the mobile phase (x_1_); temperature of column (x_2_) and flow rate (x_3_). The variations of these parameters affect chromatographic behavior of substances: changing in a composition of the mobile phase induces a variation of the degree of ionization, and column temperature as well as flow rate affects retention behavior. Retention factor of first peak (*p*-hydroxyatorvastatin; Rt *p*-ATO); last peak (atorvastatin, lactone forms; Rt ATO-l); symmetry of four compounds (Sym *p*-ATO, Sym *o*-ATO, Sym ATO, Sym ATO-l) and relative retention time of first peak (RRt *p*-ATO) were selected as responses (see Table 1 for symbols).

**Table 1:** Experimental design of the face-centered central composite design and experimentally obtained responses; x_1_-content of acetonitrile in the mobile phase, *x_2_*, temperature of column and *x_3_*, flow rate, Rt, retention time, Sym, symmetry of compounds, RRt, relative retention time.

Factor levels			Responses						
*x_1_* (%)	*x_2_* (°C)	*x_3_* (mL min^−1^)	Rt *p*-ATO	Rt ATO-l	Sym *p*-ATO	Sym *o*-ATO	Sym ATO	Sym ATO-l	RRt *p*-ATO
65	35	1	2.851	6.982	1.183	1.122	1.132	1.102	1.102
65	35	1	2.856	7.089	1.146	1.098	1.107	1.121	1.126
65	30	1	3.158	7.445	0.753	0.779	0.872	0.979	1.213
65	35	1	2.874	7.251	1.128	1.168	1.232	1.246	1.207
65	35	1	3.214	7.861	1.047	1.119	1.206	1.123	1.143
70	30	1.2	2.905	7.652	0.441	0.503	0.427	0.913	0.556
60	35	1	2.134	7.128	1.148	1.204	0.986	0.998	1.162
65	35	0.8	2.302	7.636	0.897	0.962	1.165	0.946	1.237
65	35	1	2.826	7.241	1.147	1.201	1.136	1.121	1.145
70	30	0.8	2.342	8.359	0.595	0.436	0.661	0.626	1.362
60	40	0.8	1.764	6.352	0.432	0.553	0.614	0.661	1.245
60	30	0.8	2.089	6.452	0.559	0.651	0.801	0.867	1.268
60	30	1.2	2.453	7.753	0.982	0.812	0.806	0.995	0.585
70	40	1.2	1.472	6.152	1.284	0.993	0.898	0.962	0.565
65	40	1.0	1.976	6.573	1.112	1.085	0.903	1.011	1.082
60	40	1.2	2.543	8.459	1.052	1.021	0.984	0.924	0.581
70	35	1.0	1.752	6.561	1.064	1.011	1.006	0.922	1.151
65	35	1.0	2.847	7.139	1.152	1.117	1.127	1.098	1.142
70	40	0.8	0.472	6.083	1.109	0.595	0.685	0.538	1.365
65	35	1.2	3.367	8.124	1.203	1.206	1.167	1.121	0.536

In the preliminary study, resolutions were satisfactory (Rs > 2) and were not considered as critical factors, but the problem was elution of the first peak (*p*-hydroxyatorvastatin) which was too close to the peak of the mobile phase. That is why relative retention time of the first peak was selected as response. The elution time of the last peak (ATO-l) determined the time of analysis (which is very important from a practical point of view), and was included as one of the responses for the global optimization.

The preliminary screening step was carried out according to a 2^3^ full factorial design with center point in order to identify the significant factors affecting the response. Three repetitions are generally carried out in order to know the experimental error variance and to test the predictive validity of the model. ANOVA generated for 2k Factorial design shows that curvature is significant for all the responses since the p-value is less than 0.05.

Optimum chromatographic conditions were estimated by a face-centered central composite design using Derringer’s desirability function global optimization approach. Face-centered CCD is chosen due to its flexibility and high efficiency. The design required 2k + 2k + n = 20 runs, where k is the number of parameters studied (k = 3) and n the number of central points (n = 6). Replicates of the central points were performed to estimate the experimental error.

All experiments were conducted in randomized order to minimize the effects of uncontrolled variables that may introduce a bias on the measurements. Table 1 summarizes the conducted experiments and responses. Appropriate calculations were done with the Design-Expert 7.0 software (Stat-Ease Inc. Minneapolis, MN, USA). The quadratic mathematical model for three independent factors was used to describe the response surface (Equation 1):

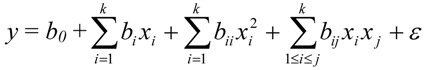
(1)
where y is the single response (Rt *p*-ATO, Rt ATO-l….) to be modeled, b is the regression coefficient, and x_i_, x_j_ represent factors. Calculated coefficients of the response model and obtained p-values (rows below) are given in Table 2.

In order to get more realistic model, insignificant terms with corresponding p-value > 0.05 at 95% confidence level, were eliminated from the model by a “backward elimination” process.

**Table 2:** Response models with p-value and statistical parameter (R^2^) obtained from ANOVA.

	b_0_	b_1_	b_2_	b_3_	b_12_	b_13_	b_23_	b_11_	b_22_	b_33_	R^2^	R^2^ Adjusted
Rt**	2.84	−0.20	−0.47	0.38	−0.38	−0.053	0.11	−0.79	−0.17	−0.099	0.9665	0.9363
*p*-ATO	0.0001 ^a^	0.0043	0.0001	0.0001	0.0001	0.4182	0.1175	0.0001	0.1429	0.3732		
Rt	7.27	−0.13	−0.40	0.33	−0.55	−0.51	0.20	−0.44	−0.27	0.6	0.9409	0.8878
ATO-l	0.0001	0.1042	0.0003	0.0014	0.0001	0.0001	0.0397	0.0121	0.0862	0.0018		
Sym **	1.14	0.032	0.17	0.14	0.18	−0.13	0.066	−0.033	−0.21	−0.089	0.9794	0.9608
*p*-ATO	0.0001	0.0834	0.0001	0.0001	0.0001	0.0001	0.0054	0.3236	0.0001	0.0187		
Sym **	1.16	−0.070	0.11	0.13	0.067	−0.020	0.080	−0.086	−0.26	−0.11	0.9799	0.9618
*o*-ATO	0.0001	0.0013	0.0001	0.0001	0.0036	0.2755	0.0012	0.0180	0.0001	0.0048		
Sym	1.16	−0.051	0.052	0.036	0.063	−0.049	0.1	−0.16	−0.27	-	0.9698	0.9426
ATO	0.0001	0.0130	0.0126	0.0632	0.0079	0.0266	0.0003	0.0006	0.0001	(0.7937)		
Sym	1.12	−0.048	−0.028	0.13	0.030	0.040	0.034	−0.14	−0.11	−0.068	0.9546	0.9138
ATO-l	0.0001	0.0165	0.1222	0.0001	0.1448	0.0593	0.1007	0.0013	0.0077	0.0600		
RRt **	1.14	0.016	−0.015	−0.37	0.005	−0.032	0.003	0.026	0.017	−0.24	0.9907	0.9823
*p*-ATO	0.0001	0.2247	0.2593	0.0001	0.7284	0.0391	0.8235	0.2831	0.4718	0.0001		

^a^ p-value.

Since the residuals of model describing RRt *p*-ATO were not distributed with constant variance, the software recommended mathematical transformation. For this purpose the Box Cox plot was used as tool which helps determination of the most appropriate power transformation that can be applied to response data. In this, case reciprocal square root (with lambda = −0.5) was recommended. The obtained statistical data are presented in Table 3.

**Table 3:** Reduced response models and statistical data obtained from ANOVA (after backward elimination).

Response	Reduced response models *	R^2^	Adj. R^2^	Pred. R^2^	Adequate precision	RSD (%)
Rt *p*-ATO	2.83 − 0.02 *x_1_* − 0.47*x_2_* + 0.38*x_3_* − 0.38*x_1_x_2_* − 0.83*x_1_^2^*	0.9454	0.9258	0.8786	26.388	7.88
Rt ATO-l	7.23 − 0.40 *x_2_* + 0.33*x_3_* − 0.55*x_1_x_2_* − 0.051*x_1_x_3_* + 0.20 *x_2_x_3_* − 0.54*x_1_^2^* + 0.50 *x_3_^2^*	0.9007	0.8428	0.7602	12.984	3.88
Sym *p*-ATO	1.13 + 0.17 *x_2_* + 0.14*x_3_* + 0.18*x_1_x_2_*-0.13*x_1_x_3_* + 0.066*x_2_x_3_* − 0.22*x_2_^2^* − 0.1*x_3_^2^*	0.9695	0.9517	0.8050	22.114	6.01
Sym *o*-ATO	1.16 − 0.07 *x_1_* + 0.11*x_2_* + 0.13*x_3_* + 0.067*x_1_x_2_* + 0.08 *x_2_x_3_* − 0.086*x_1_^2^* − 0.26*x_2_^2^* − 0.11 *x_3_^2^*	0.9772	0.9606	0.9108	22.759	5.47
Sym ATO	1.16 − 0.051 *x_1_* + 0.052*x_2_* + 0.063*x_1_x_2_* − 0.049 *x_1_x_3_* + 0.1*x_2_x_3_* − 0.16*x_1_^2^* − 0.27*x_2_^2^*	0.9564	0.9310	0.7508	19.841	6.25
Sym ATO-l	1.11 + 0.13 *x_3_* − 0.17*x_1_^2^* − 0.13*x_2_^2^*	0.8370	0.8064	0.7443	15.555	8.27
RRt *p*-ATO **	0.94 − 1.5 × 10^−3^ *x_1_* + 5.4x10^−3^*x_2_* + 0.23*x_3_* + 3.0 × 10^−3^*x_1_x_2_* + 0.015*x_1_x_3_* − 1.68 × 10^−3^*x_2_x_3_* − 0.016*x_1_^2^* − 0.11*x_2_^2^* + 0.19*x_3_^2^*	0.9960	0.9925	0.9806	45.543	1.59

* Coefficients with *p* < 0.05 are included. Factors are in coded value;** Mathematical transformation to reciprocal square root.

The qualities of the fitted mathematical models were examined by the coefficient of correlation R2. But, this coefficient always decreases when a variable is eliminated from a regression model. Because of that, the adjusted R2 (which takes the number of selected variables) was taken into account. The obtained adjusted R2 were within acceptable limits of (R2 ≥ 0.80), indicating that the experimental data were a good fit to the equations.

All the reduced models have *p* values < 0.05, implying the models were significant. “Adeq Precision” measures the signal (response) to noise (deviation) ratio and a value greater than 4 is desirable, indicating the signal is adequate and, therefore, the model is significant. The relative standard deviation (RSD) is a measure of the reproducibility of the model and a model can be regarded as reasonably reproducible if the RSD is <10% [26].

It is obvious from Table 3 that different responses were affected by different factors. The content of acetonitrile in the mobile phase (*x_1_*) is the most significant factor (the largest absolute coefficients) for retention of *para*- and *ortho*-hydoxyatorvastatin and symmetry of atorvastatin lactone. Symmetries of other peaks were affected mostly by temperature of the column (*x_2_*), while relative retention time of the para isomer was under the influence of flow rate (*x_3_*). A positive sign indicates direct correlation between response and factor, while a minus shows decreasing response with increasing factor. For example: increasing acetonitrile in the mobile phase reduces retention of ATO-l (−0.54, desirable effect), but reduces symmetries of peaks of *o*-ATO (−0.086) and ATO (−0.16) which is undesirable. Since the selected responses were not affected in the same manner, an additional optimization procedure was needed.

In order to get the best chromatographic performance, the multicriteria methodology [27,28] developed by Derringer and Suich [29] was employed. The method involves transformation of each predicted response, ŷ, to a dimensionless partial desirability function, d_i_, which includes the researcher’s priorities and desires when building the optimization procedure. There are several ways for calculating the desirability function, depending on whether each of the n responses has to be maximized or minimized, or has a target value. If the response i is to be maximized the quantity d_i_ is defined by Equation (2):

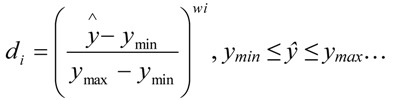
(2)

*d_i_* = 1, *ŷ* > *y_max_*; *d_i_* = 0, *ŷ* < *y_min_*

where *y_max_*, *y_max_* are the lowest and the highest values obtained for the response i, and w_i_ is the weight. Individual desirability functions di range from 0 (undesired response) to 1 (a fully desired response).

In both cases, d_i_ will vary non-linearly while approaching the desired value. But with a weight of 1, d_i_ varies linearly. In this work we chose weights equal to 1 for all responses. The partial desirability functions are then combined into a single composite response, the so-called global desirability function D, defined as the geometric mean of the different d_i_-values:

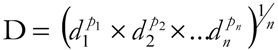
(3)
where n is the number of responses, and *p_n_* is the weight of the responses. Weight of the response is the relative importance of each of the individual functions d_i_. The relative importance *p_i_* is a comparative scale for weighting each of the resulting di in the overall desirability product and it varies from the least important (*p_i_* = 0.1) to the most important (*p_i_* = 10).

A value of D close to 1 means that the combination of different criteria is globally optimal. If any of the responses or factors falls outside their desirability range, the overall function becomes zero. The goals of multicriteria optimization for seven responses are shown in Table 4.

**Table 4:** Criteria for multivariate optimization of the individual responses.

Response	Goal	Weight	Lower limit	Upper limit	Importance
Acetonitrile	range	1	60	70	3
Temperature	range	1	30	40	3
Flow rate	range	1	0.8	1.2	3
Rt *p*-ATO	range	1	0.472	3.367	3
Rt ATO-l	target = 7	1	6.083	8.459	4
Sym *p*-ATO	target = 1	1	0.432	1.284	3
Sym *o*-ATO	target = 1	1	0.436	1.206	3
Sym ATO	target = 1	1	0.427	1.232	3
Sym ATO-l	target = 1	1	0.538	1.246	3
RRt *p*-ATO	range	1	0.856	1.366	3

**Figure 2:**
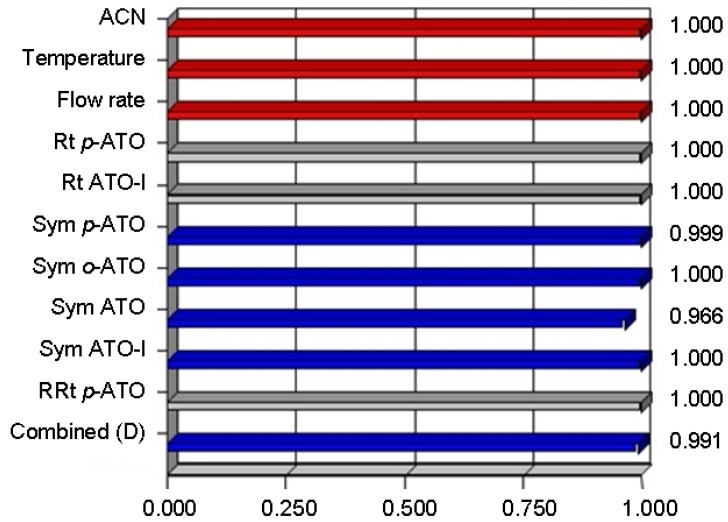
Bar graph showing individual desirability values (*d_i_*) of various objective responses.

The optimization procedure was conducted under listed conditions and restrictions. The partial desirability functions (d_i_) of each of the responses, and the calculated geometric mean as the maximum global desirability function (D = 0.919) are presented in Figure 2.

**Figure 3:**
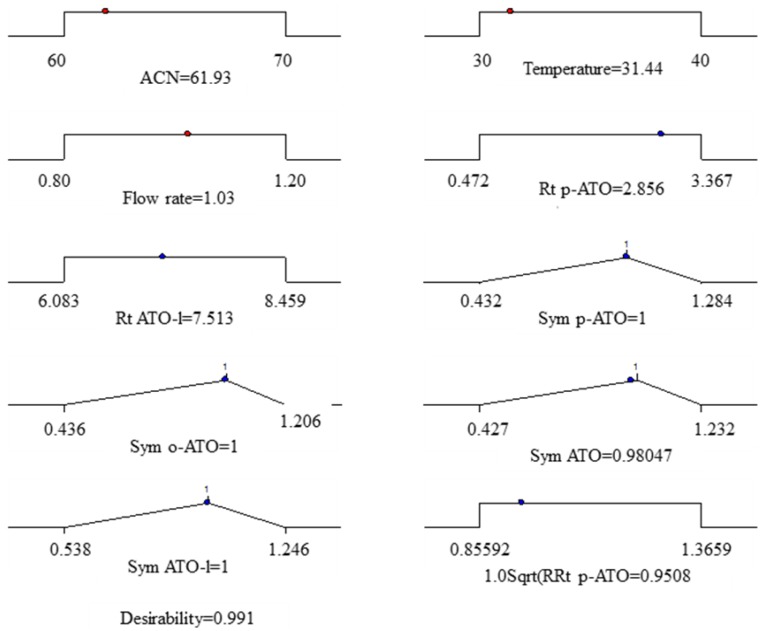
Graphical representation of the constraints accepted fot the determination of global desirabilty and obtained optimal conditions.

Desirability function calculations were performed using Design-Expert^®^ 7.0. Obtained results are graphically presented (Figure 3).

**Figure 4:**
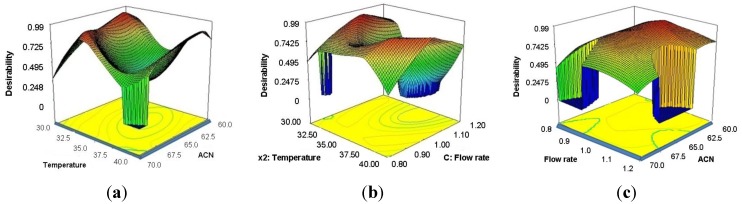
Three-dimensional graph: (**a**) *D* = *f*(temperature, ACN) with factor *x_3_* (flow rate) held at 1.03 mL min^−1^; (**b**) *D* = *f*(temperature, flow rate) with factor *x_1_* (content of acetonitrile in the mobile phase) held at 61.93%; (**c**) *D* = *f*(flow rate, ACN) with factor *x_2_* (temperature of column) held at 31.44 °C.

For better visualization of the results, the global desirability function D is presented in a form of a 3D plots (Figure 4a–c). The third factor was set up at the optimum constant level.

**Figure 5:**
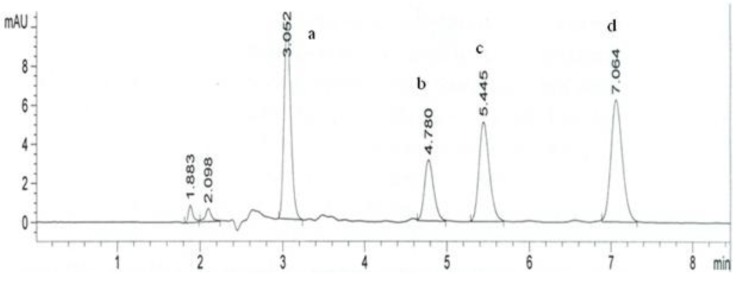
LC-PDA chromatogram of *para-*hydroxyatorvastatin (**a**), *ortho-*hydroxyatorvastatin (**b**), atorvastatin (**c**) and atorvastatin lactone (**d**) taken under optimized experimental conditions.

**Figure 6:**
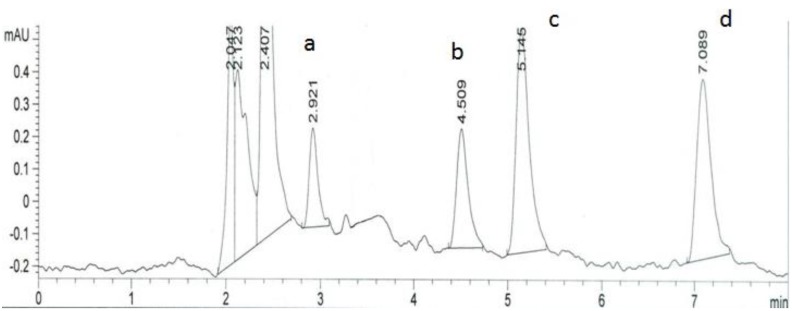
LC-PDA chromatogram of *para-*hydroxyatorvastatin (**a**), *ortho-*hydroxyatorvastatin (**b**), atorvastatin (**c**) and atorvastatin lactone (**d**) in plasma.

The coordinates related to the functions maximum are selected as the best operating conditions. The best chromatographic conditions are achieved with 61.93% of ACN, temperature of column 31.44 °C and flow rate 1.03 mL min^−1^. A representative chromatogram is shown in Figure 5. The method has been successfully applied for determination of atorvastatin (acid and lactone form) and its metabolites in plasma (Figure 6).

## 3. Experimental

### 3.1. Chemical and Reagents

Atorvastatin, *para*-hydroxyatorvastatin, *ortho*-hydroxyatorvastatin and atorvastatin lactone reference standards were donated by Nobel Ilaç (Istanbul, Turkey). Rosuvastatin, used as Internal Standard (IS) was donated by the Medicines and Medical Devices Agency (Belgrade, Serbia). HPLC-grade acetonitrile were purchased from Sigma Aldrich (Steinheim, Germany) and ammonium acetate used for preparation of buffers was purchased from Merck (Darmstadt, Germany). Ultrapure water was obtained by means of a TKA Water purification system (Niederelbert, Germany).

### 3.2. Standard Solutions

Stock solutions of atorvastatin, *o*-hydroxyatorvastatin and *p*-hydroxyatorvastatin were prepared by dissolving standards (5 mg) in a mixture of acetonitrile and water (10 mL, 90:10, v/v) and atorvastatin lactone (5 mg) in acetonitrile (5 mL). These stock solutions were divided into 0.5 mL portions and stored at −8 °C. Before using, 0.5 mL portions of all solutions (except atorvastatin lactone) were freshly diluted to 50 mL with 100 mM ammonium acetate buffer, pH 4.6 (pH was adjusted with glacial acetic acid). A 5 mL portion of atorvastatin lactone stock solution was diluted in 10 mL of buffer. The concentrations of obtained solutions were 5 µg mL^−1^. Solutions for method optimization were prepared by diluting 1 mL of stock solutions with buffer to obtain a concentration of 500 ng mL^−1^. All solutions were kept on ice to minimize conversion between lactone and acid form of atorvastatin and metabolites.

### 3.3. Biological Samples

Biological samples were obtained from Wistar rats (*Rattus rattus*). They were given atorvastatin over six weeks. Plasma samples were frozen immediately after sample withdrawal to minimize acid-lactone interconversion and stored at −80 °C. Preparation of samples was performed by solid-phase extraction, and the samples were kept on ice all time during sample preparation. The plasma samples (1 mL) were mixed with 1 mL of ammonium acetate (0.1 M, pH 4.6) and centrifuged at 1,600 *g* for 5 min. The supernatant was subsequently transferred to 1 mL C18 (100 mg) SPE cartridges (J.T. Baker, Deventer, The Netherlands), pre-conditioned with 2 mL methanol followed by 2 mL water. The cartridges were washed with 1 mL water and 1 mL methanol:water (5:95, v/v). The analytes were eluted with 1 mL methanol:ammonium acetate buffer (0.1M, pH 4.6, 95:5, v/v) and evaporated to dryness under a stream of nitrogen. The residues were reconstituted in 0.5 mL mobile phase for injection in the HPLC system.

### 3.4. Chromatographic Procedure

The HPLC analyses were done by using an Agilent Technologies 1200 Series (Santa Clara, CA, USA) chromatographic system equipped with a PDA detector, binary pumps G1312A, diode array detector G1315D, degasser G1379B and manual injector G1328B. The sample loop was 20 μL. Separations were performed on a ZORBAX Eclipse Plus C18 Analytical (4.6 mm × 250 mm, 5 μm particle size) column (Agilent) with detection at 254 nm. Experiments were prepared according to the plan given in Table 1 below. Mobile phases were degassed and vacuum filtered through a 0.45 μm membrane filter (Alltech Associates, Lokeren, Belgium).

### 3.5. Software

Experimental design, statistical analysis and desirability function calculation were performed by using MarvinSketch 5.8.2 (Chem Axon Ltd., Somerville, MA, USA, and Budapest, Hungary) and Design-Expert^®^ 7.0 (Stat-Ease Inc., Minneapolis, MN, USA).

## 4. Conclusions

The chemometric approach for optimization of chromatographic separation of atorvastatin (acid and lactone) and its metabolite has been demonstrated. The chemometric methodology chosen for the particular objectives was very successful in the retention behavior exploration.

Full factorial design was used to screen the chromatographic factors that had significant effects on the analysis time response. The significant factors were optimized by applying central composite design and surface response methodology. Since there was a mix of responses with different targets, a multi-criteria decision-making tool (Derringer’s desirability function) was applied. After defining a global desirability according to the accepted constraints, optimal chromatographic conditions were established.

The goal of this optimization of RP-HPLC was not to compete with the more potent LC/MS/MS assay with very low LOQ values. The proposed optimized RP-HPLC, as a method available worldwide, was applied to the quantitative analysis of real plasma samples, with satisfying analytical parameters. This investigation also showed that chromatographic techniques coupled with chemometric tools can provide a complete profile of a separation process, making this combined technique a powerful analytical tool.
